# Evaluation of white sandstone mechanical behaviour and the energy evolution of prepeak unloading damage

**DOI:** 10.1038/s41598-022-06680-z

**Published:** 2022-02-18

**Authors:** Zhonghao Liang, Zhuoqun Yu, Longren Guo, Sa Huang, Nan Qin, Zhijie Wen

**Affiliations:** 1 School of Mechanical and Electrical Engineering, Qingdao University of Science and Technology, Qingdao, 266061 China; 2 Key Laboratory of Mining Disaster Prevention and Control, Shandong University of Science and Technology, Qingdao, 266590 China

**Keywords:** Geology, Petrology

## Abstract

Deep high-stress roadway excavation under unloading disturbance inevitably leads to damage deterioration of the surrounding rock, which poses a serious threat to its stability. To explore the energy characteristics of white sandstone damaged by peak front unloading, uniaxial compression tests were conducted on damaged rock samples. The results show that the peak strength and modulus of elasticity of the rock sample gradually decrease with increasing damage degrees. The external work input energy, releasable elastic strain energy and dissipation energy all decreased with increasing damage. Damage evolution curves and equations of the rock samples were obtained based on the damage instantiation model established by the principle of energy dissipation and release. The effects of unloading damage on the fracture characteristics of the rock samples were analysed from both macro and microscopic viewpoints, and the results showed that a micro fracture in the rock is transformed from brittle–ductile damage, while macroscopic damage occurs in the form of a "shear"-"splitting"-"mixed shear-splitting" damage process. This paper has certain research and reference value for understanding the damage evolution characteristics of rocks with peak front unloading damage.

Energy resources (coal, metal ore, oil and gas, etc.) are an important pillar of the rapid development of the world economy. With the progress of science and technology and economic development, the demand for mineral resources and underground mining intensity are increasing, the shallow resources of the earth are decreasing and being exhausted, and underground mining is becoming increasingly larger. Kilometre-deep exploitation of energy resources has become the norm. Xie et al.^1^. As the underground depth increases, the engineering and geological conditions are also becoming more complex such that the deep mine surrounding rock structure is different from the shallow geological environment; due to the high and low stresses between the deep surrounding rock and the low intensity of the surrounding rock before excavation, it is already in a latent plasticity or plasticity state, and the mining of mineral resources is actually an unloading process for rock mass. Chen et al.^2^. The mining stress caused by the mining of mineral resources can lead to destruction of damaged surrounding rock, and further weaken its mechanical properties and surrounding rock structure. However, restricted by the characteristics of underground engineering, the mechanical properties of damaged surrounding rock and support structure must be used to form a common bearing body to control the large deformation and failure of surrounding rock. Zheng et al.^3^. Therefore, the mechanical properties of deep damaged surrounding rocks arequite different from intact rock, and the rock mechanics problems in the process of deep mineral resource mining have become the hotspots and difficulties in domestic and foreign research^4,5^. Experts and scholars at home and abroad have conducted extensive studies on rock mechanical properties under the action of unloading. The full stress–strain curve of marble was obtained by a rigid rock mechanics test machine, revealing the influence of initial unloading failure on the mechanical properties associated with the deformation and failure of the surrounding rock mass of the project^6,7^. Tunnel excavation caused a strong stress distribution in the high ground stress environment, leading to disruption of the surrounding rocks. At the same time, the influence of rock mass unloading on deformation parameters at high and low temperatures was obtained^8,9^. Research has obtained the internal mesostructure characteristics, which have a significant impact on the macroscopic damage characteristics of rock under different stress and stress paths^10–12^. According to the overall deformation characteristics of rock during the test, the damage variable of the rock is defined by a continuous factor, the principle of strain equivalence and the statistical damage theory, and the rationality of the damage variable equation of the damage process has been demonstrated. Wang et al.^13^. Rocks in underground projects such as coal mining and tunnel excavation require repeated loading and unloading, and research has focused on the energy evolution characteristics of rock under circulating load. A study found that the value of the damage variable increased with the strain index^14,15^. In essence, rock destruction is a thermodynamic process. During this process, the internal and external energy of the rock continues to transfer and transform. The macromechanical properties of rocks are closely related to the conversion of internal energy^16,17^. The failure of coal and rock is actually an instability phenomenon driven by energy. A new damage constitutive model was established based on the dissipative energy and total input energy at peak strength, and the damage evolution curve and equation of roof rock and floor rock (RCF) composite samples were obtained. Ma et al.^18^.

However, many studies have explored the damage characteristics and evolution of rock in cyclic loading–unloading from the point of view of energy. However, the abovementioned research did not consider that the surrounding rock in the fractured zone on the surface of the deep shaft tunnel is in a low surrounding pressure state, and the energy characteristics during uniaxial stress reloading of the rock in the fractured zone by the increase and transfer of peak stress formation during the recovery process have not been studied. Moreover, a damage model for the reloading of rocks from the basis of energy evolution by triaxial stress unloading damage effects has not yet been built.

This paper first introduces the mineral components of white sandstone such that the rock is quantified. A subsequent three-axis stress unloading test was carried out for pretreatment, and then a uniaxial compression test was performed to obtain the decay law of the white sandstone mechanical properties of different peaks. At the same time, the energy evolution law of white sandstone under the uniaxial compression test is proposed, which is helpful to establish the damage constitutive model of white sandstone under the unloading damage effect and uniaxial loading. Finally, the deterioration mechanism of white sandstone under prepeak stress unloading is discussed through macro and micro characteristics.

## Test pretreatment

### Test equipment and rock sample preparation

**Figure 1:**
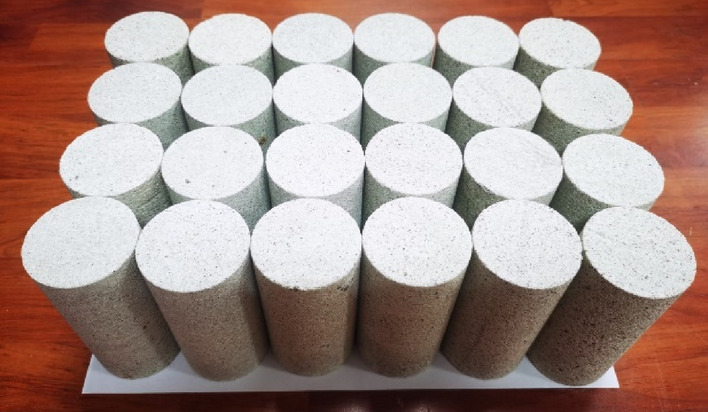
Processed white sandstone samples.

The white sandstone rock sample selected for this test is mined in the mountainous area of Zizhong County, Sichuan Province. The external structure of the rock sample is dense without obvious crack defects when using a rock core machine, stone sawing machine, and grinding machine. From a completely dense large rock block, the core type is removed, and the core sample is processed according to the requirements of the International Society for Rock Mechanics Research (ISMR). Altindag R et al.^19^ A standard cylindrical test specimen with a diameter of 50 mm and a height of 100 mm is shown in Fig. 1. The test specimen height error is $\pm$ 2 mm, and the unevenness of the two ends is $\pm$ 0.05 mm.

**Figure 2:**
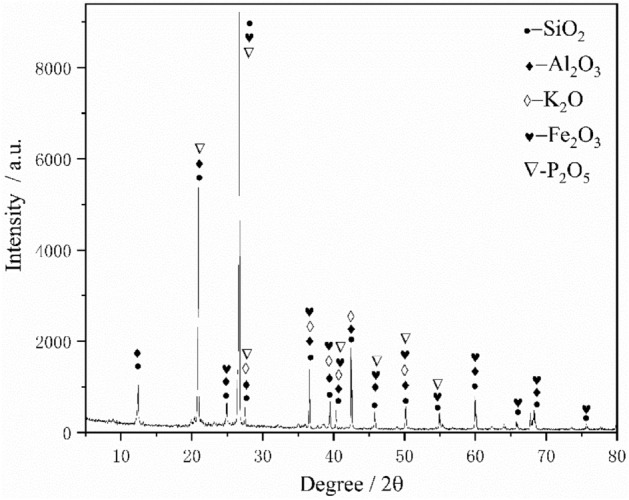
X-ray diffraction analysis.

Before the prepeak unloading damage treatment test, the test specimen was subjected to subsequent testing with the same batch of acoustic wave velocity tests to prevent excessive errors in the test data. The average density of the test specimen used was 2.30 g/cm^-3^, and the average longitudinal wave speed was 2.025 kM/s. The compound content of the X-ray spectrum (XRF) and X-dispersion spectrum (XRD) was obtained by X-ray spectroscopy, as shown in Fig. 2 and Table 1.

**Table 1:** Mass fractions of elements and corresponding oxides.

Specimen	elements		corresponding oxides	
	Compound	Conc/%	Compound	Conc/%
White Sandstone	Sr	0.011	SrO	0.004
	Al	20.772	Al_2_O_3_	24.1
	Si	67.341	SiO_2_	69.76
	P	0.825	P_2_O_5_	0.72
	Ca	0.526	CaO	0.26
	K	4.315	K_2_O	1.93
	Fe	3.518	Fe_2_O_3_	1.73

Table 1 shows that the contents of Si, Al, K and Fe in white sandstone are significantly higher than those of the other three elements, with levels of 9.76%, 24.10%, 1.93%, and 1.73%, respectively. At the same time, the oxides were determined: ${\mathrm{S}}_{\mathrm{i}}{\mathrm{O}}_{2}$, ${\mathrm{Al}}_{2}{\mathrm{O}}_{3}$, ${\mathrm{K}}_{2}{\mathrm{O}}$, and $\mathrm{Fe}_{2}\mathrm{O}_{3}$. The main components of the test specimen are quartz, clay, needle iron ore, and other substances. Among them, $\alpha - {\mathrm{S}}_{\mathrm{i}}{\mathrm{O}}_{2}$ is quartz, with a content of 69.76%; Hydrous aluminosilicate ${\mathrm{Al}}_{2}{\mathrm{O}}_{3} \cdot 2 {\mathrm{S}}_{\mathrm{i}}{\mathrm{O}}_{2}\cdot 2 {\mathrm{H}}_{2}{\mathrm{O}}$ is clay, the content of which is 24.1%. In addition, it also contains a small amount of ${\mathrm{Fe}}_{2}{\mathrm{O}}_{3}, {\mathrm{K}}_{2}{\mathrm{O}}$, etc. Among them, ${\mathrm{Fe}}_{2}{\mathrm{O}}_{3}$ is goethite, accounting for 1.73%; other substances include ${\mathrm{S}}_{\mathrm{r}}{\mathrm{O}}$, calcite accounts for only 0.004%. Therefore, the test rock specimen was determined to be quartz white sandstone; that is, the contents of quartz and various siliceous rock cuttings account for more than 95% of the total amount of sand rock cuttings, which can be obtained by X-ray diffraction (XRD) analysis, as shown in Fig. 2. Figure 2 shows multiple apparent peaks on the diffraction curve, where the intensity of the main peak reaches 9225 and the diffraction angle is 26.67°. The X-ray diffraction results further demonstrate that the main material of white sandstone is silica (SiO_2_).

### Experimental process

**Figure 3:**
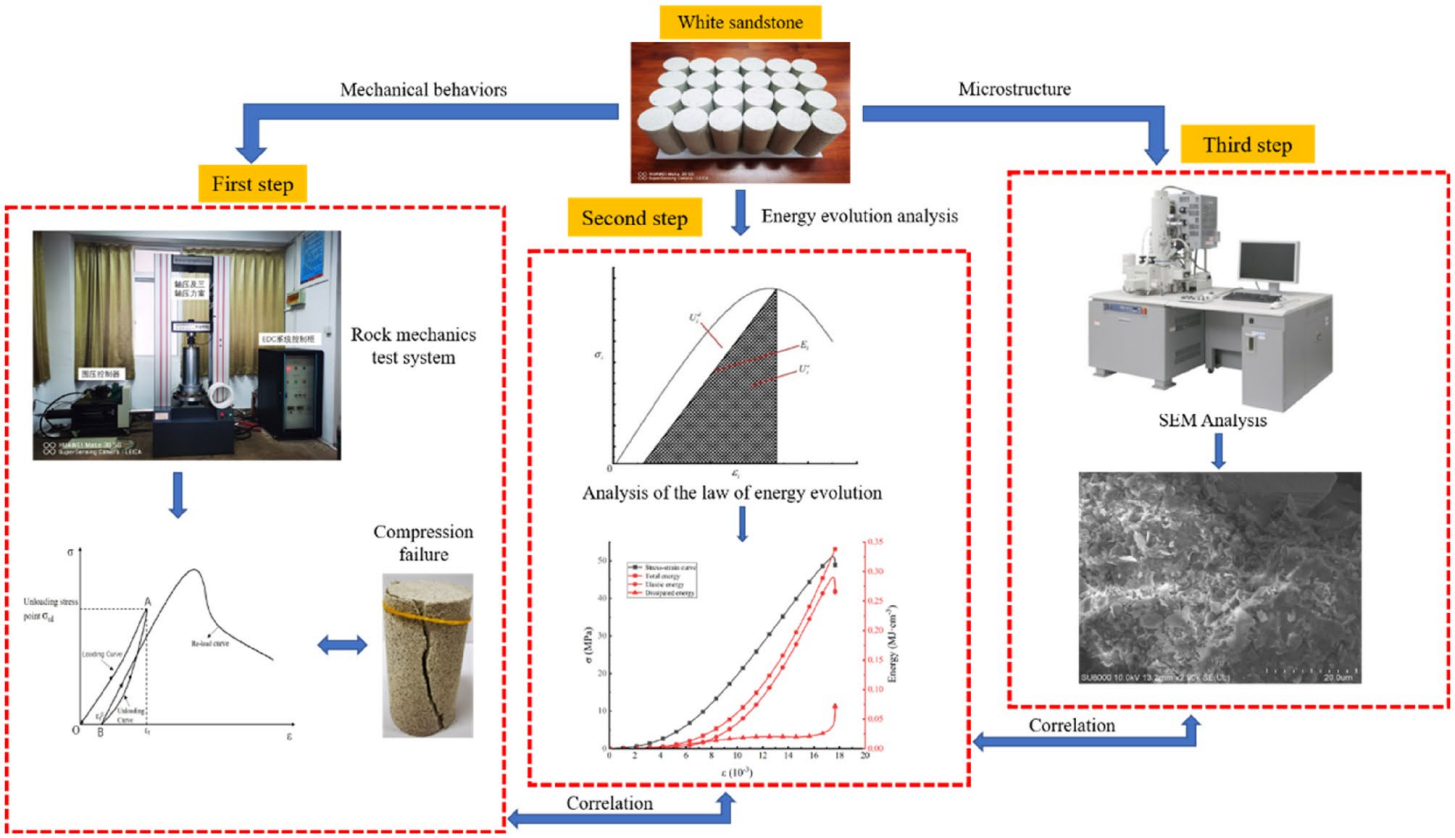
Experimental scheme for macro- and mesoscopic failure and mechanical performance testing of white sandstone.

The strong unloading disturbance during deep high stress roadway excavation inevitably leads to damage and deterioration of the surrounding rock. After roadway excavation, the stress of the surrounding rock will be redistributed, the stress state will change, and the surrounding rock will continue to be damaged until failure, which will pose a serious threat to the stability of the surrounding rock. At the same time, the available technical means do not allow for the acquisition of large and realistic samples of unloading damage in underground rock engineering. Therefore, in this test, white sandstone was selected as the test object based on the rock characteristics in previous underground engineering projects. Based on the triaxial loading unloading test, damaged rock samples were obtained. Using computer-controlled TAW-200 multifunctional material mechanics testing machine rock mechanics testing machine for uniaxial recompression test of broken rock samples, the testing machine by Changchun Chaoyang Testing Machine Factory and Qingdao University of Science and Technology jointly developed, and the failure microstructure of white sandstone was studied by scanning electron microscopy (SEM) to evaluate the influence of rock microstructure failure on macromechanical properties after unloading damage and to provide a theoretical basis for the stability evaluation of engineering construction demonstrations. The experimental process is shown in Fig. 3.

## Results and analysis

### Prepeak unloading rock stress–strain curve

**Figure 4:**
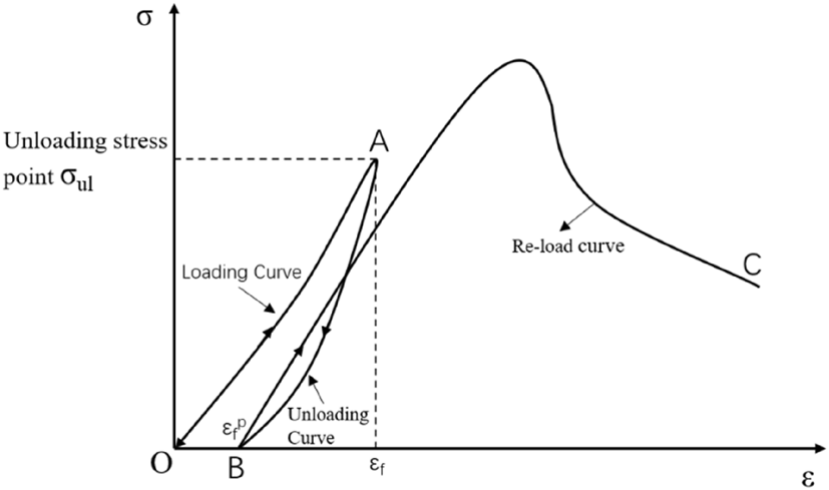
Schematic diagram of prepeak unloading damaged rock sample preparation and the reloading test.

Regarding sample preparation and the reloading test before the peak, the test can be divided into two phases (Fig. 4). First, an offloading damaged test specimen are prepared by axial pressure and a peripheral unloading method based on the intact rock-like three-axis compression experiment, and then the peak-to-load damage reloading test is carried out.

The first stage is the triaxial compression test of the complete rock sample (see the OAB section in Figs. 4 and 6a). The specific steps are as follows: 1. First, Martin point out that after the rock load is loaded to 40% of the peak strength^17,20^, and damage is generated. Unloading tests were conducted based on peak compressive strength tests of intact white sandstone at a specific peritectic pressure (σ3 = 1 MPa), and the unloading points were determined to be ${\sigma }_{ul}=0, 50\%, 60\%, 70\%, 80\%,\, \mathrm{and}\, 90\%$ before the peak and cumulative for five working conditions. Prepeak unloading test requirements: simultaneous unloading of axial pressure and confining pressure, an axial unloading rate of 1.0 MPa/s, and an unloading rate of confining pressure of 0.05 MPa/s. This method can ensure that the test specimen is always in the stress state of the surrounding rock during the unloading process and prevent the test specimen's bearing capacity from being suddenly reduced due to the excessively fast unloading of the confining pressure and subsequent failure. 2. The test can be stopped when the axial load is unloaded to 2 kN. The indenter on the testing machine is always in contact with the test specimen, which prevents the test specimen from escaping the restraint and leads to excessive data dispersion.

The second stage is the prepeak unloading and reloading test of the damaged specimen (section BC in Figs. 4 and 6b). The test method is the same as the conventional uniaxial compression test, the loading method is displacement loading, and the loading rate is $0.2\,\mathrm{ mm}/\mathrm{min}$. The purpose is to obtain the stress–strain curves of specimens with different damage degrees and provide basic data for analysis of the mechanical characteristics of prepeak unloading damaged rock samples.

**Figure 5:**
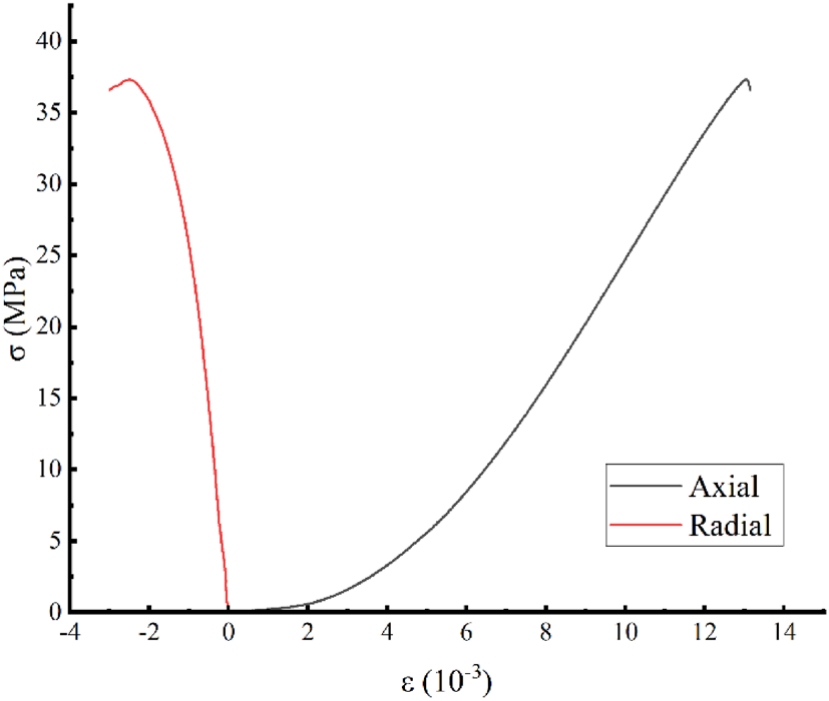
Nondestructive rock sample stress–strain curves.

Figure 5 shows that the stress–strain curve bends slightly upwards in an upward concave shape at lower stresses during the non-destructive rock sample re-load test. As the stress increases to a certain value, the curve gradually becomes straight, followed by an instantaneous fall, with no obvious yielding phase, exhibiting plastic-elastomeric rock properties.

**Figure 6:**
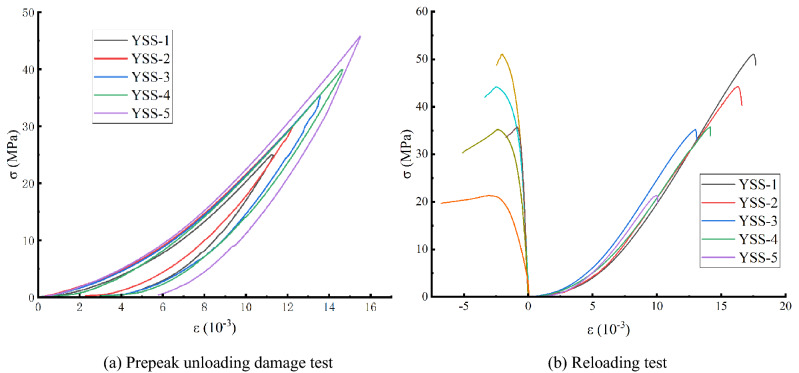
Stress–strain curves of the prepeak unloading damage and the reloading test (σ3 = 1 MPa).

Figure 6a shows that in the prepeak unloading damage experiment, during the loading process of the white sandstone specimen, due to the internal structure adjustment of the specimen and the compaction and closure of the cracks, the overall stress–strain curve presents an upwards concave shape. However, the unloading rebound deformation has a hysteresis phenomenon, and at the same time, irrecoverable residual deformation occurs, which is caused by closure, slippage, and displacement of the internal structural surface of the rock sample when subjected to external load stress. However, the loading and unloading curves of different grades are all open-loop curves, and with increases in the stress level, the residual deformation of the rock sample gradually increases, and the area of the open-loop curve gradually increases, that is, the accumulated energy inside the rock sample gradually increases. At the same time, the prepeak unloading damage variable is calculated by the elastic modulus method^21^, which can more accurately obtain the damage variable of the prepeak unloading damage test by coupling plastic deformation and the damage mechanism:

1 $$ D = 1 - \left(1 - \frac{{\varepsilon^{r} }}{\varepsilon }\right)\frac{{E^{r} }}{{E_{0} }} $$

**Figure 7:**
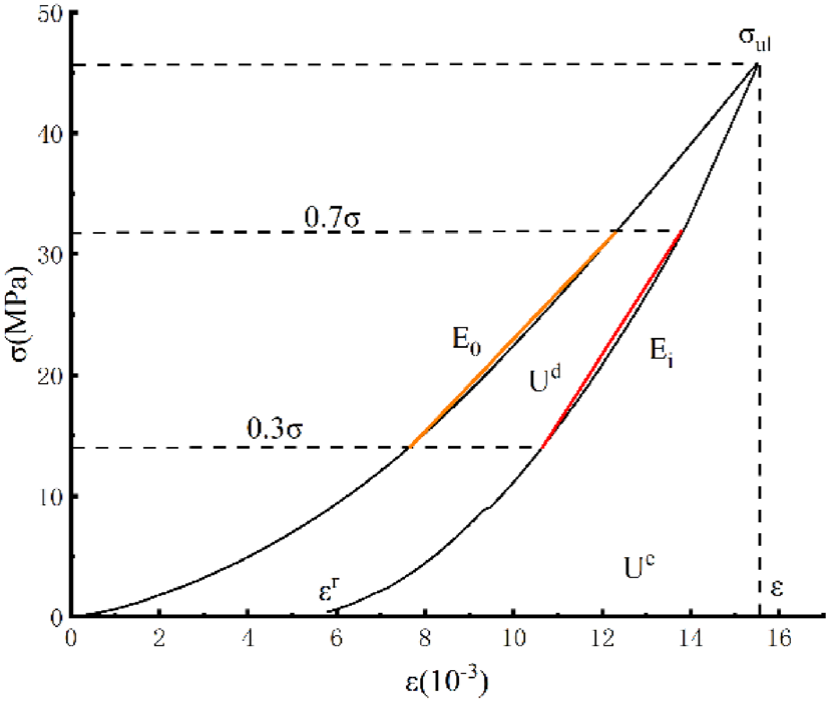
Parameter value diagram.

In this formula, $D$ is the variable of the peak unloading damage, $\varepsilon^{r}$ is the residual strain, $\varepsilon$ is the total strain, ${E}_{i}$ is the unloading flexible modulus, and ${E}_{0}$ is the initial elastic modulus. The modulus of the elastic modulus ranges from 30 to 70% in this paper^22^. The selection of each parameter is shown in Fig. 7, and the specific test values are shown in Table 2. The calculated damage variable and residual strain conversion curve are shown in Fig. 7, where the unloading point $\sigma_{ul}$ is the unloading strength and the peak intensity ratio.

**Table 2:** Mechanical parameters of the damaged rock mass.

Serial number	σ_ul_/MPa	ε^r^/%	ε/%	E_i_/GPa
YSS-1	50	0.003	0.011	4.49
YSS-2	60	0.0034	0.012	4.21
YSS-3	70	0.0041	0.014	4.15
YSS-4	80	0.0048	0.015	3.88
YSS-5	90	0.0057	0.016	3.54

Figure 6b gives the white sandstone stress–strain curve from the single-axis reloading test in the prepeak unloading damage experiment, and the overall reloading curve can be divided into four stages of rocks: 1. Compaction stage. When the rock test specimen is in a low stress state, the test specimen stress–strain curve exhibits an upper concave shape, and the original fracture surface of the test specimen is gradually closed. At the same time, at this stage, the tester shows small radial displacement, and the total test specimen volume gradually decreases as the stress increases. The white sandstone test specimen was damaged before the initial peak, and the internal structure was damaged; thus, the deformation in this stage was more obvious. 2. Linear elastic deformation stage . When the stress increases to a certain stage, the curve is approximately straight, and the microelastic fissure in the test specimen is stable in this stage. 3. Unstable fracture development stage. At this time, the test specimen is transformed by elasticity into plasticity. In this stage, the development of microruptures in the test specimen shifts to a mass change, resulting in test specimen failure. The volumetric strain and rate are rapidly increased, and the volume changes from compression to expansion. 4. Destruction stage. When the test specimen bearing capacity reaches peak stress, internal fracture of the test specimen rapidly occurs, and cross penetration facilitates the formation of a macroscopic fracture surface. Then, deformation is mainly manifested as slipping along a macroscopic surface, and the test specimen carrier is rapidly lowered. The complete test specimen is relatively straight, with no obvious yield stage, showing plastic-elastic rock properties. In addition, when ${\sigma }_{ul}=50\%\sim 80\%$ after unloading injury, the test specimen was approximately S-type, and then the moment dropped, showing the properties of plastic–plastic rock, and when $\sigma_{ul} \ge 80\%$ after unloading injury, the curve of the test specimen was significantly slowed after the peak because with the increase in damage, the native microfilm gap in the rock sample continuously developed, and the inner native damaged structure surface produced slip, friction, etc. This phenomenon causes a sharp increase in the rock direction of the rock sample such that the volume of the rock sample transitions from volume compression to volume expansion in a short period. As the degree of damage increases, the compressed volumetric variables of the rock sample when unloading is loaded and the solar termination of the rock sample gradually decrease earlier than the expansion phenomenon. The peak stress–strain curve is transformed from the upper concave-S type, and the lateral radial strain speed is relieved from the moment fall–slowly fall. The rock exhibits a trend towards a transition from brittle damage to ductility, from "plasticity-elastic rock properties transition to "plasticity—elastic—plasticity rock properties".

**Figure 8:**
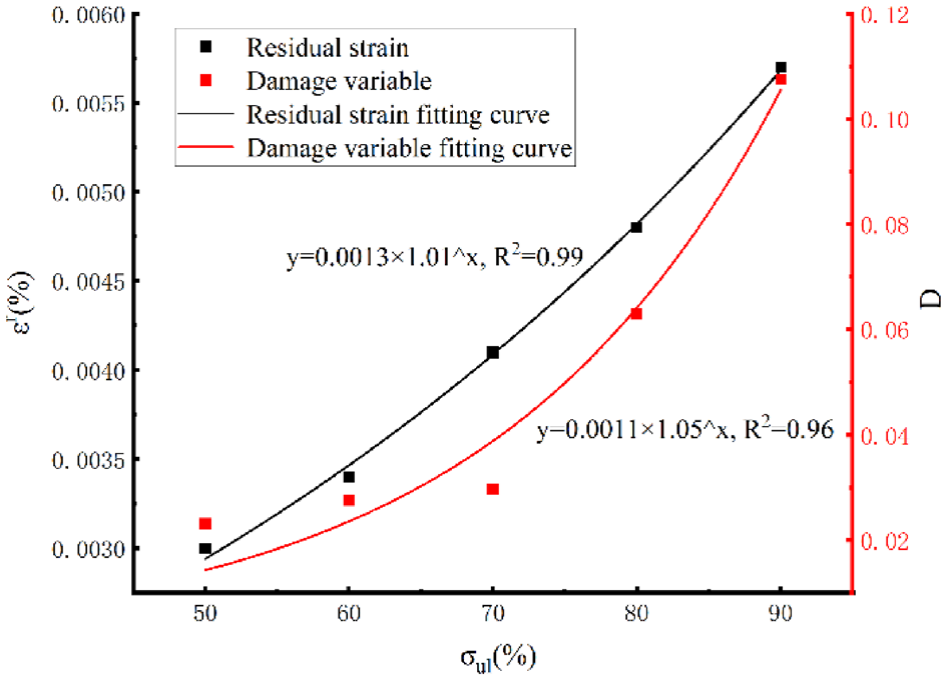
Variation in damage variables and residual strain with the unloading point.

Figure 8 and Table 2 show that the damage variables and residual strain are positively correlated with the change in the unloading point; with the increase in the unloading point, the damage variable is gradually increased from 0.003 to 0.0057, and the residual strain increases to 0.003 and 0.0034, respectively. In contrast, as the unloading point increases, the variation trend of the two is substantially consistent, and the fitting curve meets the index variation^8^.

**Figure 9:**
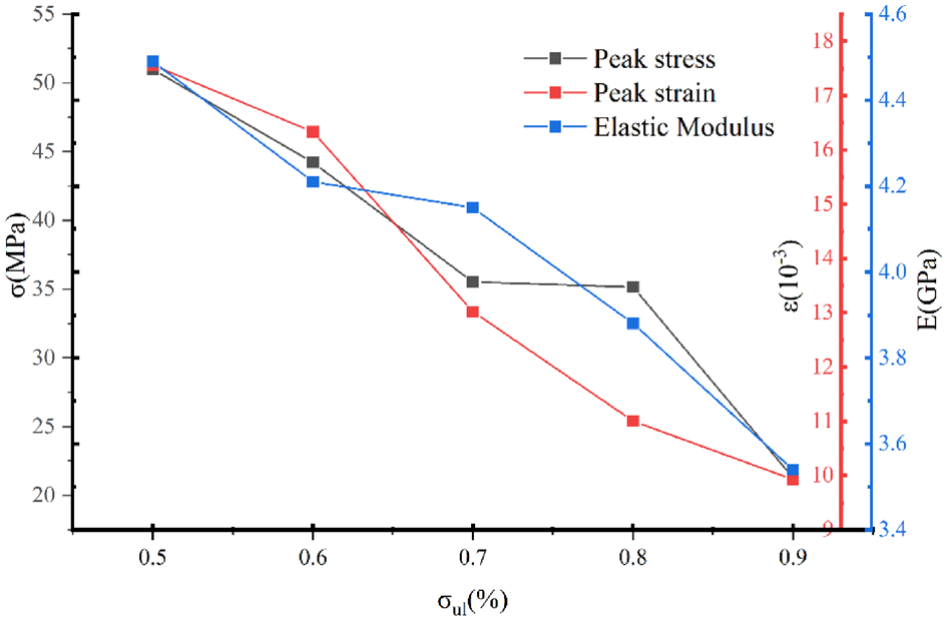
Variation curve of reloading deformation characteristics with the unloading point.

Figure 9 shows that the elastic modulus, peak stress, and strain of the specimen have the same changing trend, and they all decrease with increasing damage. When $\sigma_{ul} \le 80\%$, unloading is loaded, the stress–strain curve of low-damage rock samples exhibits brittle failure, and the stress decreases in the postenter peak; however, when $\sigma_{ul} \ge 80\%$, the unloading–reloading curve increases with the degree of damage, and the peak stress drop is significantly reduced. The radial strain has a significant plastic damage character. In summary, the overall strength of the white sandstone test specimen has a decrease in overall strength, the degree of damage in the structural surface of the test specimen is intensified, the fissure is continuously developed, and the elastic modulus is lowered.

### Energy evolution analysis of rock failure

According to the law of irreversible thermodynamics, energy conversion is an essential feature of the physical process of matter, and material destruction is a phenomenon of state instability driven by energy^23^. In the process of rock deformation and failure, a certain internal relationship exists between energy dissipation, energy release and rock deformation and failure. Rock deformation and failure are caused by the combined effect of energy dissipation and energy release. Therefore, the damage variable in this paper starts from the basic principles of energy dissipation and release, combined with the elastic modulus and strain parameters in the actual stress–strain curve, and gives an apparent damage variable suitable for describing the damage behaviour of elastoplastic materials, which can more accurately describe the damage and deformation of white sandstone damaged by prepeak unloading.

When the unit volume of the rock specimen is damaged and deformed under external force, if the physical process of the specimen does not have any heat exchange with the outside world, then the whole system is considered a closed system. At this time, the total input energy generated by the external force's work can be known from the first law of thermodynamics:

2 $$ U{ = }U^{e} + U^{d} $$

where ${U}^{d}$ is the unit dissipation energy and ${U}^{e}$ is the unit release elastic strain energy.

**Figure 10:**
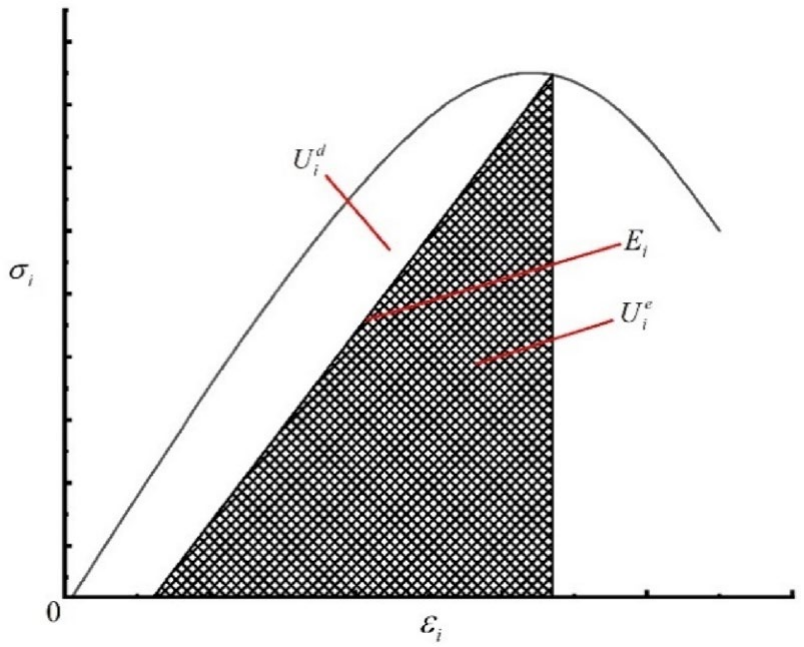
Rock unit stress–strain curve.

Figure 10 is a rock body unit stress–strain curve, and an area of a unit ${U}_{i}^{d}$ dissipation can be used to form internal damage of a unit, resulting in deterioration and loss of rock sample mechanical properties. This change process complies with entropy growth, that is, the microthermodynamic process. The increment is always greater than 0. The shaded area ${U}_{i}^{e}$ can release elastic strain energy, which is the internal release of elastic strain energy in the unit. ${E}_{i}$ is the unloading of the elastic modulus.

The above analysis shows that the energy absorbed by the uniaxial condition is the area contained below the rock sample stress–strain curve, that is, the axial stress–strain curve is available. Considering that the curve equation is unknown, a fixed integration is used.

3 $$ U{ = }\int_{{0}}^{{\varepsilon_{{1}} }} {\sigma_{{1}} } d\varepsilon_{1} = \sum\limits_{i = 1}^{n} \frac{1}{2} \left( {\sigma_{i + 1} + \sigma_{i} } \right)\left( {\varepsilon_{i + 1} - \varepsilon_{i} } \right) $$

4 $$ U^{e} = \frac{1}{2}\sigma_{1} \varepsilon_{1} = \frac{{\sigma_{1}^{2} }}{{2E_{i} }} $$

5 $$ \varepsilon_{i}^{e} = \frac{1}{{E_{i} }}\left[ {\sigma_{i} - \nu_{i} \left( {\sigma_{j} + \sigma_{k} } \right)} \right] $$

At this time, the average values $\overline{E}$ and $\overline{\nu }$ can be determined from the uninstalling test determined by rock uniaxial cycle compression.$\varepsilon_{i}^{e}$ is the total flexible strain in the three main stress directions. Formula (4) is the calculation formula for the releasable elastic strain energy of prepeak unloading damaged rock mass elements suitable for engineering applications, selecting the uninstall elastic modulus ${E}_{i}$ and Poisson's ratio $\nu$ from 30 to 70% of the stress–strain curve^22^.

The energy loss of the lower rock body unit under complex stress is satisfied with the following relationships.

6 $$ U^{d} = U - U^{e} $$

**Figure 11:**
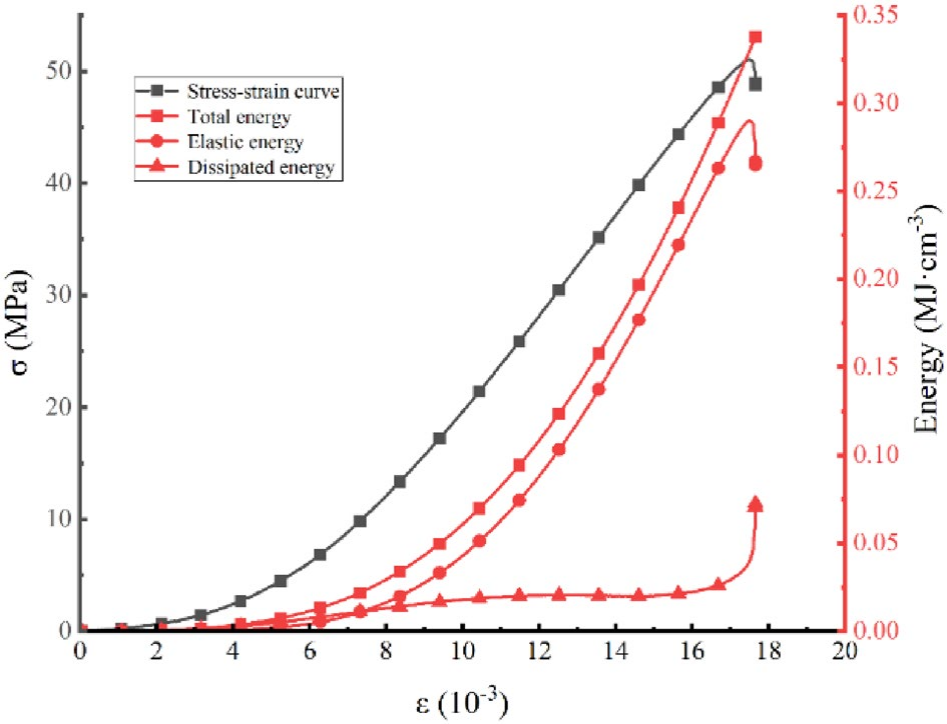
Stress–strain curves of rock samples as a function of energy.

**Figure 12:**
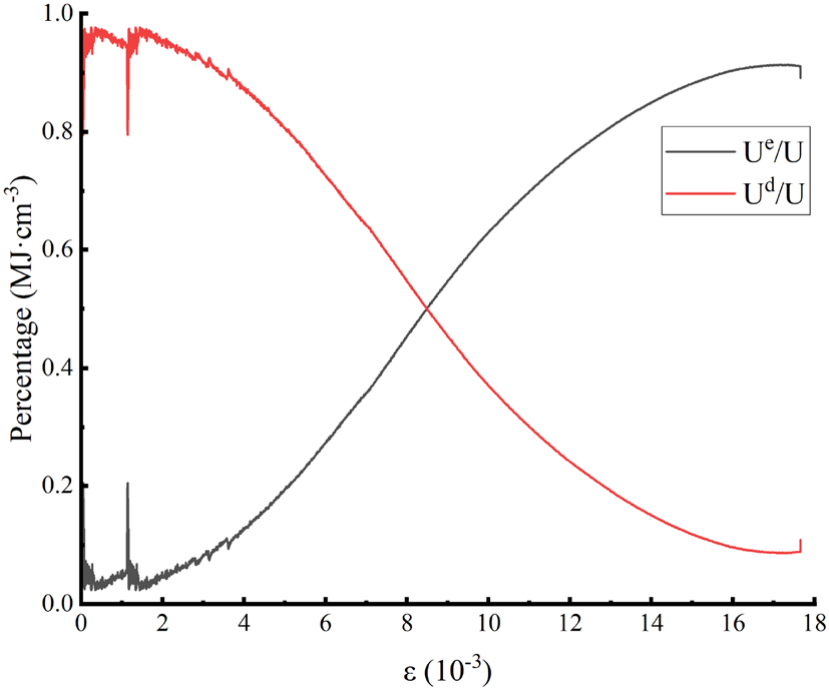
Relationship between elastic energy, dissipative energy share and strain in rock samples.

Formulas (3), (4), (5), and (6) are calculated to obtain Table 3, and the data in Table 3 can be consolidated. Figure 11 and Fig. 12 can then be obtained; Fig. 11 shows the 50% stress and then the rock sample. Figure 12 shows the relationship between the elastic properties and dissipation energy, which is proportional to the strain after $\sigma_{ul} = 50\%$ is unloaded. As shown in Fig. 11, the energy of the external force is substantially converted during test specimen reloading to elastic energy storage in the rock internal structure, the total energy and elasticity of the test specimen have a parabolic growth trend, and the two curves are basically complicated. The initial growth rate of internal dissipation can remain gentle. However, in the fracture stage after the peak of the test specimen, the elasticity in the internal structure of the test specimen is stored on the surface of the test specimen, which is rapidly increased, resulting in failure of the test specimen. As shown in Fig. 12, the elastic energy ratio rapidly increases during loading to $\varepsilon = 8.7 \times 10^{ - 3}$, the dissipative energy is sharply reduced, and it linearly changes. In the $\varepsilon = 8.7\sim 14 \times 10^{ - 3}$ section, the change is logarithmic. When $\varepsilon \ge 14 \times 10^{ - 3}$ for time player performance, the dissipation energy is basically unchanged after the maximum point is reached, and after the short-term stability is experienced, the elasticity can be dissipated due to the sharp decrease in elasticity due to the damage to the test specimen, which can increase sharply.

**Table 3:** Peak front unloading damage to white sandstone reloading energy parameters.

Serial number	σ_ul_/MPa	U/MJ·cm^-3^	U^e^/ MJ·cm^-3^	U^d^/ MJ·cm^-3^
YSS-1	50	0.34	0.29	0.05
YSS-2	60	0.29	0.23	0.06
YSS-3	70	0.19	0.15	0.04
YSS-4	80	0.18	0.16	0.02
YSS-5	90	0.07	0.064	0.006

**Figure 13:**
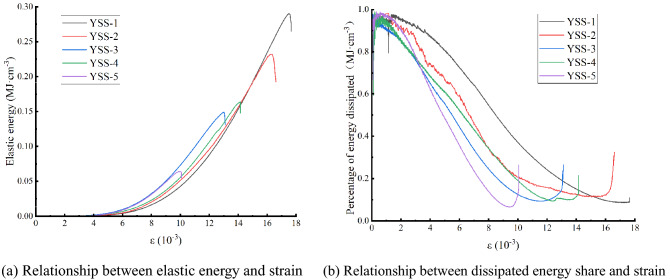
Evolution of reloading energy versus strain in prepeak unloading damaged white sandstone.

Figure 13 shows the energy evolution curve of reloading damaged rock samples before peak unloading. As shown in Fig. 13a, the elastic energy evolution curve travels substantially along a particular function curve, and as the increased elasticity of the strain increases, the elasticity can be regarded as the current stress loading level in the loading process. The damage level has nothing to do, and the result is the same as that in the literature^24^. However, with increasing damage degree, the elastic energy accumulated before peak failure decreases gradually. Figure 13b shows that the evolution law of dissipated energy is opposite to that of elastic performance. The overall trend is that the dissipated energy decreases with increasing damage degree and strain.

The amount of energy damage of the rock mass unit is defined as:

7 $$ D_{E} = \frac{{U^{d} }}{{U^{c} }} $$

8 $$ D_{E} = \frac{{U^{d} }}{{U^{c} }} = 1 $$

In this formula, $U^{c}$ is the critical energy dissipation value when the unit strength is lost. Material parameters, regardless of the stress state, can be measured by rock mechanical tests.

Equation (9) is based on energy dissipative rock mass cell strength loss criteria.

At the same time, according to concrete and rock damage mechanics^25^, the rock damage state equation can be expressed as:

9 $$ Y = \frac{{U^{e} }}{{1 - D_{E} }} $$

In this formula, Y is the conjugate variable of the damage energy and dissipation rate with D_E_.

The evolution of rock damage can also be calculated from the theoretical formula, and the two can be compared. Rock damage evolution equation^25^ is:

10 $$ D_{E} = 1 - \exp \left[ { - B\left| {Y - Y_{0} } \right|^{\frac{1}{n}} } \right] $$

In this formula, B, n, and Y_0_ are all material parameters of the rock depending on the basic material properties of the rock.

Initially, no damage to the rock samples was assumed in the early stage of the reloading test. When $Y_{0} = 0$, two consecutive logarithms on both sides of Eq. (11) are taken to obtain:

11 $$ \ln \left[ { - \ln \left( {1 - D} \right)} \right] = \ln B + \frac{1}{n}\ln Y $$

Let:

12 $$ \left\{ \begin{aligned} &y = \ln \left[ { - \ln \left( {1 - D} \right)} \right] \hfill \\& x = \ln Y \hfill \\ \end{aligned} \right. $$

Therefore, Formula (13) becomes a linear relationship curve.

13 $$ y = ax + b $$

By reloading the experiment, the known test data can be entered into Eq. (13), the test data of X and Y can be obtained, the linear fit is achieved by the linear regression determination coefficient, and x and y have a linear relationship. If the linear correlation is strong, the coefficients $a, b$ are calculated by $B, n$:

14 $$ n = \frac{1}{a} $$

15 $$ B = e^{b} $$

**Figure 14:**
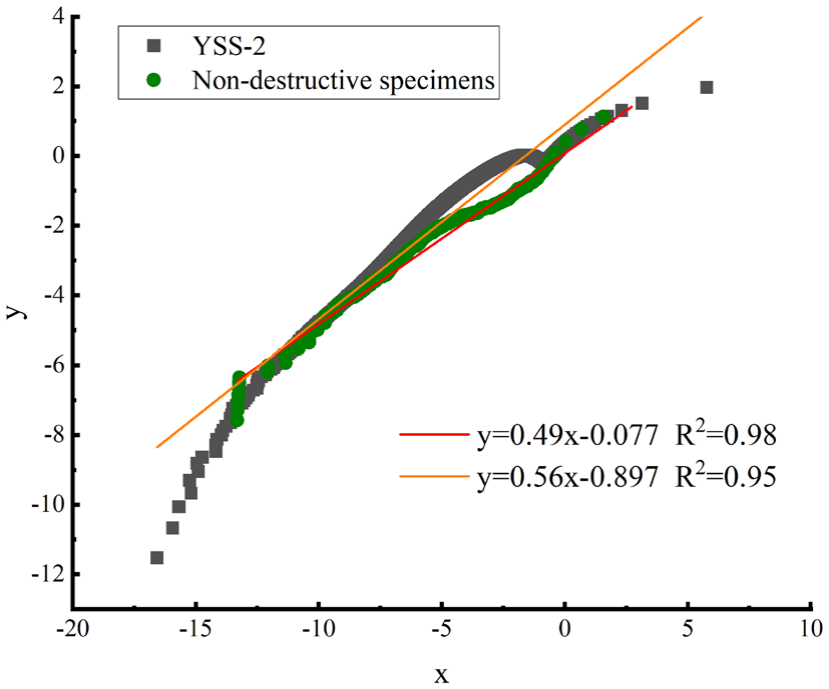
Linear fitting of the damage evolution equation of white sandstone specimens.

The nondestructive test specimen data and $\sigma_{ul} = 60\%$ unloaded time data were entered into the above formula to obtain Fig. 14. Figure 14 shows the linear fit result of a peak-free unloading damaged rock sample and a nondestructive rock, and the results show that the linear correlation is extremely high, that is, the linear correlation of x and y is reasonable, and the rock damage evolution equation meets the experimental results.

**Figure 15:**
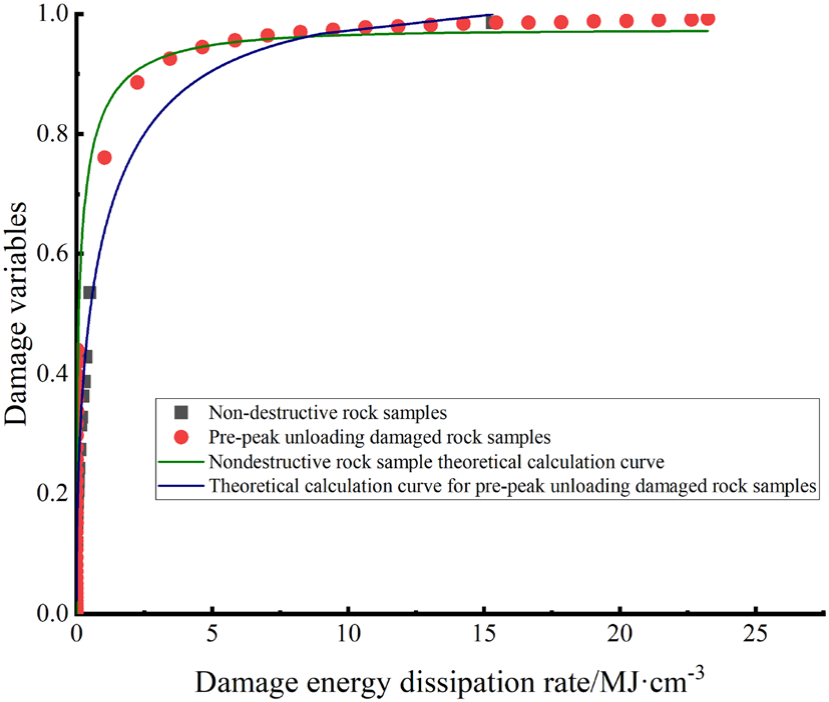
Damage evolution equation curve of white sandstone specimens.

Figure 15 shows the theoretical evolution equation and experimental results of lossless rock samples and prepeak unloading damaged rock samples. The theoretical damage evolution equation can show the evolution of damage during the recarrying process of the white sandstone test specimen after the peak, and Fig. 15 shows that the rock sample is removed before the peak is removed, resulting in internal generation of the test specimen. Mechanical behaviour such as many microporous closures and internal structural surface slip friction consumes a large amount of energy, accelerating the damage to rock samples, which is consistent with the previous conclusions.

### Failure and Deterioration Mechanism of White sandstone

The internal structure of the study material usually involves the scale problem, that is, the length range of the research space. Rock material research mainly occurs at the following three scales: the microscopic scale (10^–6^ m), mesoscale (10^–4^ m), and macroscopic scale (10^–2^ m).

**Figure 16:**
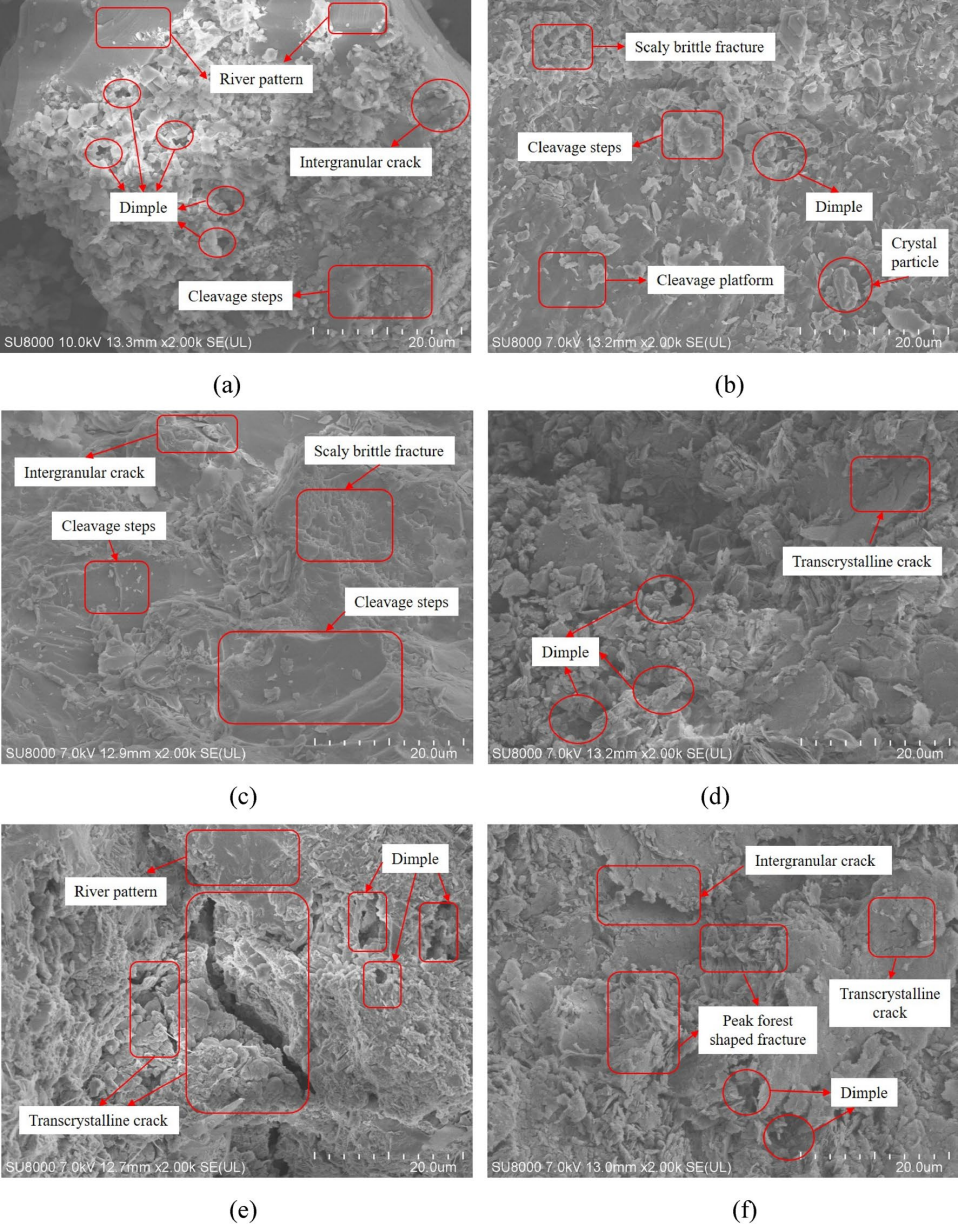
Prepeak unloading damaged white sandstone meso-failure structure (×2000): in the (**a**) nondestructive state, (**b**)–(**f**) increase with the unloading damage level to 50%, 60%, 70%, 80%, and 90%, respectively.

This article is based on the scope of constructive structural characteristics, the reloading fracture mechanism of white sandstone under prepeak unloading is studied from the meso point of view, semistructure analysis adopts the Hitachi High-tech SU8000 Scanning Electronic Microscope, the abovementioned test disruption of the test specimen is separately evaluated by an electron microscope scan, yielding a fracture scan image at 2000 magnification, and the SEM image of white sandstone fracture with varying damage is shown in Fig. 16. As shown in Fig. 16, as the degree of unloading injury increases, the fracture mode of the fine structure in the white sandstone is significantly changed.

1. Fig. 16a–f shows a white sandstone nondestructive test specimen and an unloading damage test specimen at 2000 × magnification for fracture scanning electron microscopy. The fracture surface of the test specimen is obvious. The obvious decision step is obvious. The fracture surface is relatively flat, and a clear and small river pattern is evident along the fracture direction of the decision step. When the white sandstone test specimen has been damaged after $\sigma_{ul} = 50\%$ unloading injury, the observation surface has a significant decompression platform, the fracture observation surface is relatively flat, only a small number of crystalline debris particles are evident, and a plurality of decline is noted in the platform. Follow the steps. When $\sigma_{ul} = 60\%$ unloading injury occurs, the fracture observation surface is a relatively obvious scale brittle fracture, crystal cracks are observed, and many decay steps are generated. After $\sigma_{ul} = 70\%$ unloading damage, the micromorphology of the fracture begins to change from brittle to ductile fracture. Dimple groups are obvious on the observation surface, and the whole fracture surface is coarser than the observation surface of the upper-level fracture. After $\sigma_{ul} = 80\%$ unloading damage, obvious transgranular cracks are generated in the low concaveness of the fracture, and many dimple groups are distributed on the observation surface. After $\sigma_{ul} = 90\%$ unloading damage, many crystal particles gather in the low recess of the fracture, and the transgranular crack and intergranular crack cross each other.
2. During the loading process of white sandstone after prepeak unloading damage, when the degree of unloading damage is low, the friction between crystal particles on both sides of white sandstone fracture is small, the relative displacement between crystal structures is relatively stable, an obvious cleavage step morphology is easy to produce, and when it moves relatively between crystal structural planes, due to the incongruous deformation of crystal structure, stress concentration occurs, resulting in an obvious river pattern. When the degree of unloading damage is high, due to the existence of defects in the crystal structure, the free energy in the structure spontaneously transitions to a lower energy, the dislocation phenomenon increases rapidly, the strength of the crystal particles decreases markedly, and cracks develop from the internal dislocation to form transgranular cracks. At the same time, due to the large unloading damage, the cohesion between structural planes is substantially reduced, and the friction increases inversely. The cracks in the internal structure of the specimen develop rapidly. The fracture observation surface shows the microfracture morphology of the cross connection of transgranular cracks and intergranular cracks. Due to the increase in the internal friction of the specimen, many fine crystal debris particles produced by friction between crystal particles gather at the fracture.
3. With the increase in the prepeak unloading damage degree, the internal crack development of the white sandstone specimen is more complex, and the mesostructure damage is intensified. Due to the weakening of local performance, the overall performance is more "soft", and the spalling phenomenon after rock failure is serious.

As shown in Fig. 18, the white sandstone in the nondestructive state exhibits split stretching failure. As the degree of unloading damage increases, the form of white sandstone gradually changes, such as "splitting"-"single slant cutting destruction"-"split-shear mixed destruction".

**Figure 17:**
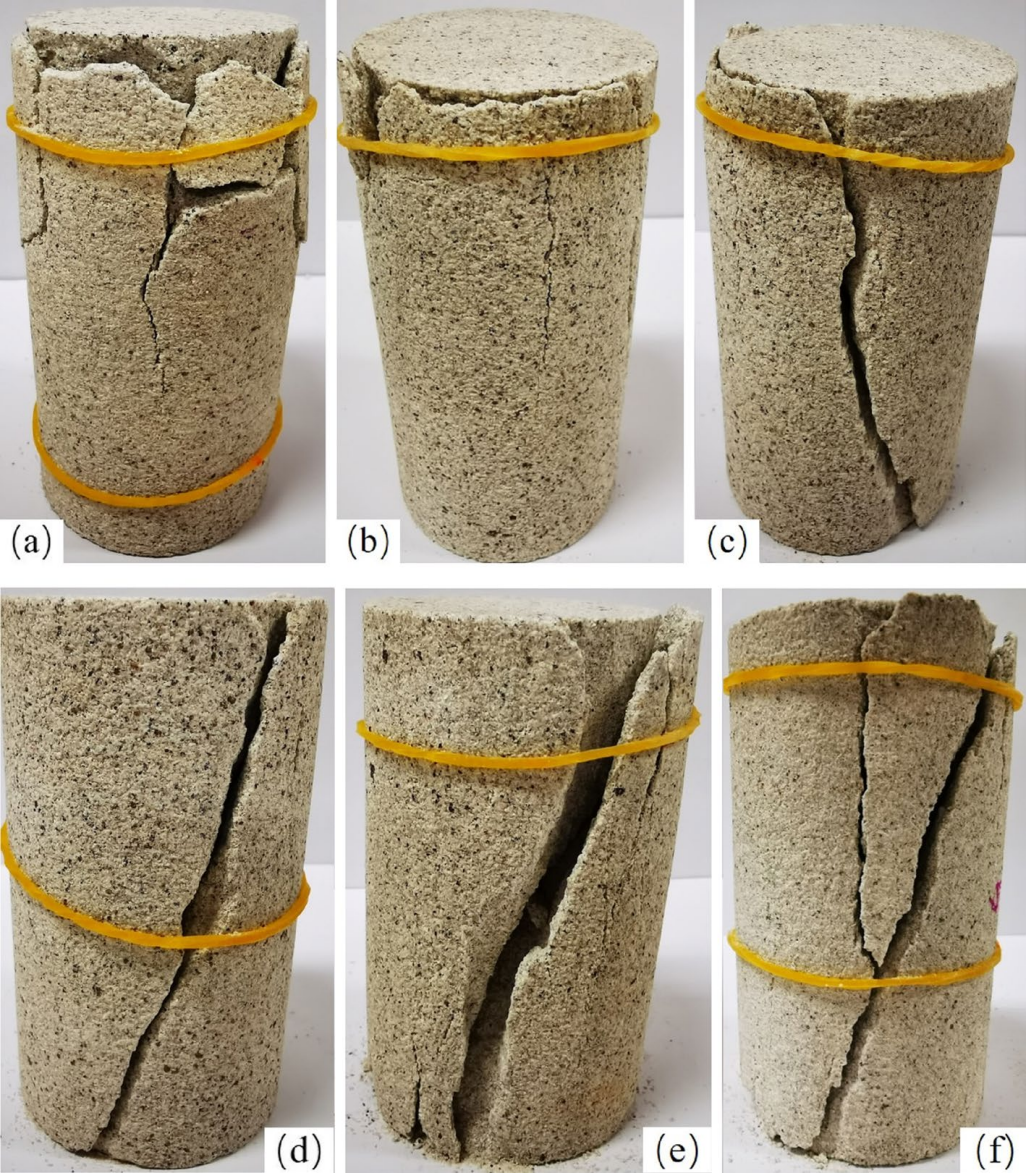
Macroscopic failure characteristics of white sandstone with prepeak unloading damage: (**a**) nondestructive state; (**b**)–(**f**) prepeak unloading damage degrees of 50%, 60%, 70%, 80%, and 90%.

As shown in Fig. 17a, the starting point of the test specimen rupturing the top generates a plurality of cracks and leads to the generation of secondary cracks. When $\sigma_{ul} = 50\%$ unloading, destruction of the sample in the form of splitting failure causes the failure surface tensile stress to exceed its tensile strength, resulting in an end portion of the specimen, which undermines the expansion. When the white sandstone specimen $\sigma_{ul} = 60\%$ is unloaded, destruction of the sample in the form of single bevel shear failure occurs, a test specimen occurs throughout the main crack from top to bottom, and the fracture angle is 68°, while several new cracks at the bottom are not obvious. When the white sandstone specimen $\sigma_{ul} = 70\%$ during unloading, destruction of the sample occurs in the form of single bevel shear failure, a main shear crack throughout the specimen, and a shear angle of 71°, the overall shape of the specimen remains substantially intact. When the white sandstone specimens $\sigma_{ul} = 80\%$ still showed significant shear failure when unloading the specimen, the primary shear fracture crack angle was 72°, and a plurality of minor cracks in the rock sample was noted top and bottom, leading to an interaction between the two fragmentation larger specimens. When the white sandstone specimen $\sigma_{ul} = 90\%$ undergoes unloading and destruction of the sample in the form of shear-splitting mixed failure, the main crack angle is 70°. In addition, splitting cracks eventually converge from the top of the shear crack, and the crack is produced at the top of the secondary small specimens. As shown in Fig. 17, the loss when unloading the specimen $\sigma_{ul} = 50\%$ due to the internal crack with a large number of native cracks easily leads to transverse tensile stress in the external force of the specimen and transverse tensile stress in the specimen when the stress exceeds the tensile limit, thus creating cleavage destruction. After unloading $\sigma_{ul} = 80\%$ rock pores and uneven development of cracks, a plurality of rock samples shows tensile fracture damage. With the increase of the degree of damage before the unloading peak due to internal white sandstone specimens unloading producing significant local defects, thus rebearing process, local defects gradually expanded, and the shear surface formed was weak, eventually leading to shear failure. Defects while damage caused local white sandstone stress become more prominent and trigger a second local connection crack fracture, eventually eroding the rock sample separation with the best form of weak in-plane shear failure.

**Figure 18:**
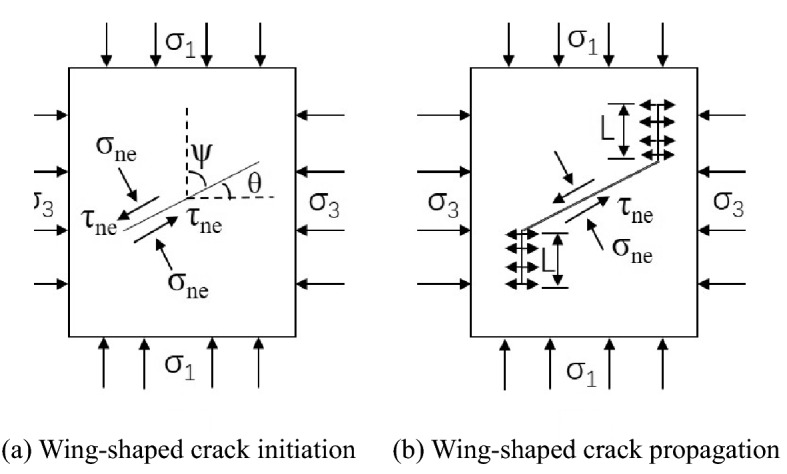
Sketch of wing crack seeding and propagation in the compressive-shear stress state.

Under loading, the rock cracked tip stress intensity factor $K_{1}$ is larger than the rock fracture toughness $K_{1C}$, and a wing crack is generated in the main stress direction at the pulley tip. Many trials and data show that the crack fissure is perpendicular to the maximum tensile stress; thus, it is expanded according to the type I crack, as shown in Fig. 18. The method and tangential stress transmitted on the crack surface are as follows (the pressure stress is positive):

16 $$ \sigma_{{{\text{ne}}}} = \sigma_{1} \sin^{2} \psi + \sigma_{3u} \cos^{2} \psi  $$

17 $$ \tau_{ne} = \frac{{\sigma_{1} - \sigma_{3u} }}{2}\sin 2\psi $$

18 $$ \sigma_{3u} = \sigma_{3} - \vartriangle \sigma $$

In this formula, $\sigma_{{{\text{ne}}}}$ and $\tau_{ne}$ are the normal stress and tangential stress on the crack surface, respectively; σ_1_ is the maximum principal stress; σ_3_ is the minimum principal stress; σ_3u_ is the minimum principal stress after unloading; ψ is the angle between the crack surface and maximum principal stress direction; and $\vartriangle \sigma$ is the unloading amount of the minimum principal stress σ_3._

According to the maximum circumferential positive stress theory, the initial crack is expanded in the circumferential direction, and the opening of the cracked angle θ = 70.5°, the punched tip stress intensity factor is:

19 $$ K_{1} = \frac{2}{\sqrt 3 }\tau_{e} \sqrt {\pi a} = \frac{2}{\sqrt 3 }\left( {\tau_{e} - \sigma_{ne} f} \right)H\left( {\tau_{e} } \right)\sqrt {\pi a} $$

20 $$ H(\tau_{e} ) = \left\{ \begin{gathered} 1,\tau_{e} = \tau_{ne} (\mu > 0) \hfill \\ 0,\tau_{ne} - \sigma_{ne} \left( {\mu \le 0} \right) \hfill \\ \end{gathered} \right. $$

In summary, the damaged rock deformation is mainly divided by cleavage, and the four modes of damage to the rock are breaking, shear destruction, shear-splitting mixing and destroying, and due to peak unloading damage, the rock internal structure is exacerbated, eventually leading to the softening characteristics of the rock structure. The test results of Fig. 17 may also indicate that new cracks will promote the development of native cracks under unloading damage, and the development of rock internal crack "damage causes cracks, further carrying pressure, and further crack increase". The last rock interior cracks are connected to each other, accelerating the breakage of the rock. This study has certain theoretical reference value for engineering practice, and the location, form, and expansion direction of the internal microcracks of rock samples require further research.

## Conclusion

In this paper, the mechanical and energetic properties of white sandstone samples damaged by peak front unloading are investigated through uniaxial compression reloading tests. The damage evolution equation is based on the evolution of the total input energy and dissipation energy at the peak strength.

1. The uniaxial compressive strength, modulus of elasticity and peak strain of the white sandstone decreased with increasing unloading damage, and the degradation of mechanical parameters increased with the obvious effect of unloading damage. At the same time, prepeak unloading damage exacerbates the structural damage within the rock, ultimately leading to a more "flimsy" rock structure as a whole.
2. As the degree of unloading damage increases, the internal structural surfaces of the rock are damaged and frictional slip occurs, the strength of the crystal structure itself is reduced, and cracks develop from within the crystal, while weakening of the crystal material properties of the rock leads to localised nascent crystal penetration cracks, internal cracks are staggered and penetrated, and then the macroscopic damage shows: the overall structure is loose, fractures are richly developed, the surface of the specimen is heavily exfoliated, and the damage exhibits a gradual evolution of "shear"—"splitting"—"mixed shear-splitting damage".
3. With the increase in the degree of unloading damage, the specimen elastic energy shows a general trend of decreasing along a specific curve, and the elastic energy accumulated at the peak damage is reduced. The dissipation energy also follows the same pattern. Based on the energy dissipation angle to establish the rock damage evolution equation, the overall exponential function of the equation trend, and the use of test results to analyse the relevant parameters, the results show that the theoretical damage variable-energy dissipation rate curve is highly similar to the test curve and the closest to the prepeak unloading damage specimen curve. Of course, this paper focuses only on the damage instantiation model and its validation under uniaxial loading of rock samples with prepeak unloading damage, and the applicability of this instantiation model to rock and its completeness require further investigation. In addition, a complete destruction constitutive model considering the prepeak stress unloading and postpeak reloading processes and field application of the model will be studied in our future work.

## References

[1] Xie HP, Ju Y, Ren S (2019). Theoretical and technological exploration of deep in situ fluidized coal mining. Front. Energy..

[2] Yang J, Yongming Y, Xi Z (2017). Topological representation of the porous structure and its evolution of reservoir sandstone under excavation-induced loads. Thermal Sci..

[3] Lujing Z, Yujun Z, Yafei H (2021). Deformation mechanism and support technology of deep and high-stress soft rock roadway. J. Adv. Civil Eng..

[4] Song S, Liu J, Yang D (2019). Pore structure characterization and permeability prediction of coal samples based on SEM images. J. Nat. Gas Sci. Eng..

[5] Tutluoğlu L, Öge F, Karpuz C (2015). Relationship between prefailure and postfailure mechanical properties of rock material of different origin. Rock Mech. Rock Eng..

[6] Hudson JA, Crouch SL, Fairhurst C (1972). Soft, stiff and servo-controlled testing machines: a review with reference to rock failure. J. Elsevier.

[7] Zhang H, Wang L, Li J, Deng H, Xu X, Chen X (2021). Mechanical properties of sandstones under initial unloading damage. Adv. Civil Eng..

[8] Zhu Z, Zihan Z, Liyuan Y (2020). Research on acoustic emission characteristics of marble damaged by prepeak unloading. IOP Conf. Ser. Earth Environ. Sci..

[9] Vermeer PA (1984). NonAssociated plasticity for soils, concrete and rock. Phys. Dry Granular Media..

[10] D. P S. Discussion of Z.T. Bieniawski's paper Mechanism of brittle fracture of rock—parts I and II. *Int. J. Rock Mech. Min. Sci. Geomech. Abstracts*.**2**, 7. 10.1016/0148-9062(70)90014-8 ..

[11] Lemaitre J, Chaboche JL, Maji AK (1993). Mechanics of solid materials. J. Eng. Mech..

[12] Yao H, Jia S, Li H (2018). Experimental study on failure characteristics of schist under unloading condition. Geotech. Geol. Eng..

[13] Wang C, Zhan S-f, Xie M-z, Wang C, Cheng L-p, Xiong Z-q, Mobayen S (2020). Damage characteristics and constitutive model of deep rock under frequent impact disturbances in the process of unloading high static stress. CompLex.

[14] Liu XS, Ning JG, Tan YL (2016). Int. J. Rock Mech. Min. Sci..

[15] Zhu A, Liu J, Wu Z, Wang L, Liu H, Xiao F, Deng C, Baraldi D (2021). Energy dissipation and damage evolution characteristics of salt rock under uniaxial cyclic loading and unloading tension. Adv. Civil Eng..

[16] Xie HP, Peng RD, Ju Y (2004). Energy dissipation analysis in the process of deformation and failure of rock. Chin. J. Rock Mech. Eng..

[17] Ren H, Zhu Y, Wang P (2020). Experimental study on mechanical characteristics of unloaded damaged white sandstone before peak. Arabian J. Geosci..

[18] Ma Q, Tan Y, Liu X (2020). Effect of coal thicknesses on energy evolution characteristics of roof rock-coal-floor rock sandwich composite structure and its damage constitutive model. Compos. Part B..

[19] Altindag R, Güney A (2005). ISRM suggested method for determining the shore hardness value for rock. Int. J. Rock Mech. Min. Sci..

[20] Martin CD, Chandler NA (1994). The progressive fracture of Lac du Bonnet granite. Int. J. Rock Mech. Min. Sci. Geomech. Abstracts.

[21] Ju Y. Applicable conditions of damage definition based on strain equivalence hypothesis. *J*. *Chin. J. Appl. Mech.*. **1**(15), 3–49. 10.1088/0256-307X/16/12/001 (1998).

[22] Meng Q, Zhang M, Han L (2018). Acoustic emission characteristics of red sandstone specimens under uniaxial cyclic loading and unloading compression. Rock Mech. Rock Eng..

[23] Mikhalyuk AV, Zakharov VV (1997). Dissipation of dynamic-loading energy in quasi-elastic deformation processes in rocks. J. Appl. Mech. Tech. Phys..

[24] Li SG (2019). Experimental study on effect of loading rate and initial damage on energy evolution of sandstone. J. Min. Saf. Eng..

[25] Xie HP (1990). Rock and concrete damage mechanics.

